# The Diagnosis of Throat and Nose Cases at a School Clinic

**Published:** 1912-03-16

**Authors:** 


### THE SCHOOL CLINIC.

### I. The Diagnosis of Throat and Nose Cases at a School Clinic.

In the organisation of a clinic for the treatment of school children suffering from diseases of the nose and throat, and who are referred by the school inspector, several considerations must be borne in mind. The school inspector will have done no more than make a rough sortage of the cases which may need treatment for enlarged tonsils and adenoids. A few other diseases sometimes occur, but the main part of the work is for these only. The doctor at the clinic must make a more accurate diagnosis with a view to deciding which children need operation and which other treatment. The clinic should therefore be organised to deal with diagnosis with non-operative treatment and with operative treatment.

### Division of Cases.

Of the children referred a minority only would need any operation if adequate other treatment were obtainable. The cases fall under three heads, those where there is increase in the lymphoid tissue around the naso-pharynx sufficient in degree to cause some obstruction to respiration; those where there is a temporary enlargement, or cases where the child is recently recovering from a common cold during the course of which the lymphoid tissue has compensatorily enlarged; and those where the symptoms of cough and of making a noise at night are due to some other disease: these are cases more or less marked of dyspepsia, the mucous disease of Dr. Eustace Smith.

Every child must be seen at the clinic in order to decide under which of these three categories he should be put. But it is often impossible to decide between the first two classes of cases by one examination, and therefore many cases must be seen two or even three times before a correct diagnosis can be made. Again, enlarged tonsils are often accused of being the cause of repeated common colds; but there is another aspect which is often lost sight of—namely, that the hypertrophy of the lymphoid tissue may be a natural reaction to combat these frequently recurring attacks which in their turn are caused by some other condition. And there is a condition present in the mouth of nearly every child that is seen which may as easily give rise to repeated colds as may an enlarged tonsil. We refer to some disease of the teeth.

Frequently the teeth are carious, or an early condition of pyorrhœa exists, short of this a mere deposit of food and tartar on the gums from want of care may be present. The treatment of these dental conditions is the first step in the treatment of the pharyngeal condition. Further, it is frequently a stage in the diagnosis of the latter. Considered, therefore, from the diagnostic point of view only, no clinic can be efficient that does not afford opportunities (*a*) for seeing the cases frequently, (*b*) for obtaining treatment for carious teeth. The latter is bound up with the separate institution of clinics for the treatment of dental cases, and will not be further considered here. But the former raises some additional practical points. The progress of these cases depends very largely upon subjective conditions rather than objective.

### The School Teacher's Duty.

Adenoids are not removed simply because they are there, but because they cause obstruction to the naso-pharynx or Eustachian tube. That is to say, the mere attendance of the child is insufficient. Some adult must be present to inform the medical officer of the progress of the case. Now it is clear that the parent cannot be expected to attend repeatedly with the child to report on the latter's condition. It may be essential and is only fair that the mother should attend the first time, but on subsequent occasions the school teacher would probably be a better person to report on the progress of the case. He or she is probably more intelligent, more observant, and better able to judge the change in the child.

These arguments bring us to the conclusion that the single clinic does not cover the whole ground. To such a clinic the children may be brought the first time by the parent; at it notes of the case should be taken, and a thorough examination made. At it also any operative work may be done, and special cases might come back there again. But the vast majority of cases with slight enlargement of tonsils and adenoids which need some treatment, but not operation, would be best seen a second time at the separate schools.

This would not entail loss of attendances every fortnight, the medical officer would be sure of the child being present, and the teacher would report as to whether the deafness, oral respiration, or other prominent symptoms had improved. The notes taken at the central clinic would be sent to the school, and the teacher would be informed which children he was to keep an eye upon and the particular points to be noticed.

